# Incomplete meniscal healing in early second-look arthroscopy does not indicate failure of repair: a case series

**DOI:** 10.1007/s00264-023-05868-w

**Published:** 2023-06-23

**Authors:** Konrad Malinowski, Dong Woon Kim, Marcin Mostowy, Przemysław Pękala, Nicholas I. Kennedy, Robert F. LaPrade

**Affiliations:** 1 Department of Anatomy, Jagiellonian University Medical College, International Evidence-Based Anatomy Working Group, Kraków, Poland; 2 Artromedical Orthopedic Clinic, Antracytowa 1, 97-400 Bełchatów, Poland; 3 Orthopedic and Trauma Department, Veteran’s Memorial Teaching Hospital in Lodz, Medical University of Lodz, st. Żeromskiego 113, 90-549 Lodz, Poland; 4 Faculty of Medicine and Health Sciences, Andrzej Frycz Modrzewski Kraków University, Kraków, Poland; 5 Twin Cities Orthopedics, Edina, MN USA

**Keywords:** Meniscus, Anterior cruciate ligament, Longitudinal meniscal tear, All-inside meniscal repair, Suture hooks

## Abstract

**Purpose:**

To assess if incomplete meniscal healing during second-look arthroscopy at six to eight weeks after all-inside suture hook meniscus repair results in longer-term failure of repair in patients with restored knee stability.

**Methods:**

From 2008 to 2013, 41 patients with post-traumatic, longitudinal, vertical, complete meniscal tears with concomitant ACL injury were treated via a two-stage surgical procedure and prospectively evaluated. In the first stage, all-inside meniscus repair was performed using suture hook passers and non-absorbable sutures. In total, there were 26 medial and 16 lateral meniscus tears. A second-stage ACL reconstruction, performed six to eight weeks later, served as an early second-look arthroscopic evaluation of meniscal healing. Clinical follow-up was performed at a minimum of 24 months.

**Results:**

Second-look arthroscopy revealed 31 cases (75.6%) of complete and ten cases (24.4%) of incomplete meniscal healing. Two patients were lost prior to follow-up, and three were excluded due to recurrent instability. Therefore, 36 patients were assessed at the final follow-up. All patients with complete meniscal healing during second-look arthroscopy achieved clinical success at follow-up. Six out of nine (66.7%) of patients with incomplete meniscal healing during second-look arthroscopy achieved clinical success at follow-up (*p* = 0.012). One saphenous neuropathy occurred (2.4%).

**Conclusion:**

Incomplete meniscal healing during early second-look arthroscopy after all-inside meniscal repair using suture hook passers and non-absorbable sutures did not necessarily result in longer-term failure in patients with restored knee stability. The described method of meniscal repair was associated with a low rate of symptomatic re-tears and complications.

## Introduction

Post-traumatic meniscal tears are commonly seen in young patients as a result of acute, sports-related trauma. These tears are typically longitudinal and vertically oriented along the peripheral third of the meniscal body and are commonly associated with knee instability due to concomitant anterior cruciate ligament (ACL) deficiency [1, 2]. In post-traumatic meniscal tears, arthroscopic repair of the injured meniscus should always be considered as the treatment of choice [3–5]. Modern arthroscopic techniques of meniscal repair can be divided into three categories: (1) inside-out, (2) outside-in and (3) all-inside [6]. The outside-in and inside-out repair techniques carry the increased risk of neurovascular complications [7]. In this regard, the all-inside repair technique offers several advantages: preservation of the meniscus blood supply and avoidance of an additional incision [8]. There are two methods of all-inside technique: one using sutures, and the other using meniscal fixation devices. Suture repair, as opposed to meniscal fixation devices, preserves the physiological mobility of the meniscus with respect to the surrounding tissues [9] and has been reported to yield better meniscal healing rates in patients with concomitant ACL tears when assessed during second-look arthroscopy [10]. Furthermore, meniscal fixation devices carry the risk of suture loosening, chondral injury and synovitis [11]. For these reasons, the all-inside suture technique, although technically demanding, can optimize patient outcomes while minimising the risk of complications [6, 12].

Successful meniscal repair depends on a successful healing which is based on two fundamental principles: solid mechanical fixation and the biological healing process [3]. The meniscal healing process progresses through a series of inflammatory, proliferative and remodelling phases that result in the formation of a functional scar [13]. Therefore, successful healing of the repaired meniscus can be assessed among the others by the fulfilment of the meniscal suture sites with scar tissue. This fulfilment can be directly visualised and assessed, i.e. during second-look arthroscopy. However, despite our current knowledge of the meniscal healing process, it is still up for debate whether the surgeon should intervene in the case of prolonged or incomplete healing, particularly when evaluated early after the repair.

The aim of this paper was to assess if incomplete meniscal healing during second-look arthroscopy at six to eight weeks after all-inside suture hook meniscal repair results in longer-term failure of the repair in patients with restored knee stability.

## Materials and methods

Ethical approval was obtained from the district medical chamber (approval number K.B.-7/2023). The study was designed in compliance with the Helsinki Declaration.

This was a retrospective cohort study of consecutive patients presenting to a single centre with post-traumatic, complete, vertical, longitudinal tears of the outer third of the meniscus and a concomitant anterior cruciate ligament (ACL) injury. During the period from 2008 to 2013, 41 consecutive patients (aged 18 to 45 years old, 24 male and 17 female) who underwent a two-stage surgical procedure were included in the study, and all data were prospectively collected into a database. Exclusion criteria were (1) meniscal body degeneration, (2) multi-ligament instability and (3) chondral injury > stage II according to the International Cartilage Research Society (ICRS) classification [14].

**Fig. 1:**
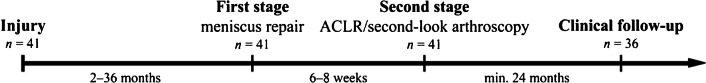
Study timeline diagram

As summarised in the study timeline diagram (Fig. 1), the first stage was performed 2 to 36 months after the initial injury.

**Fig. 2:**
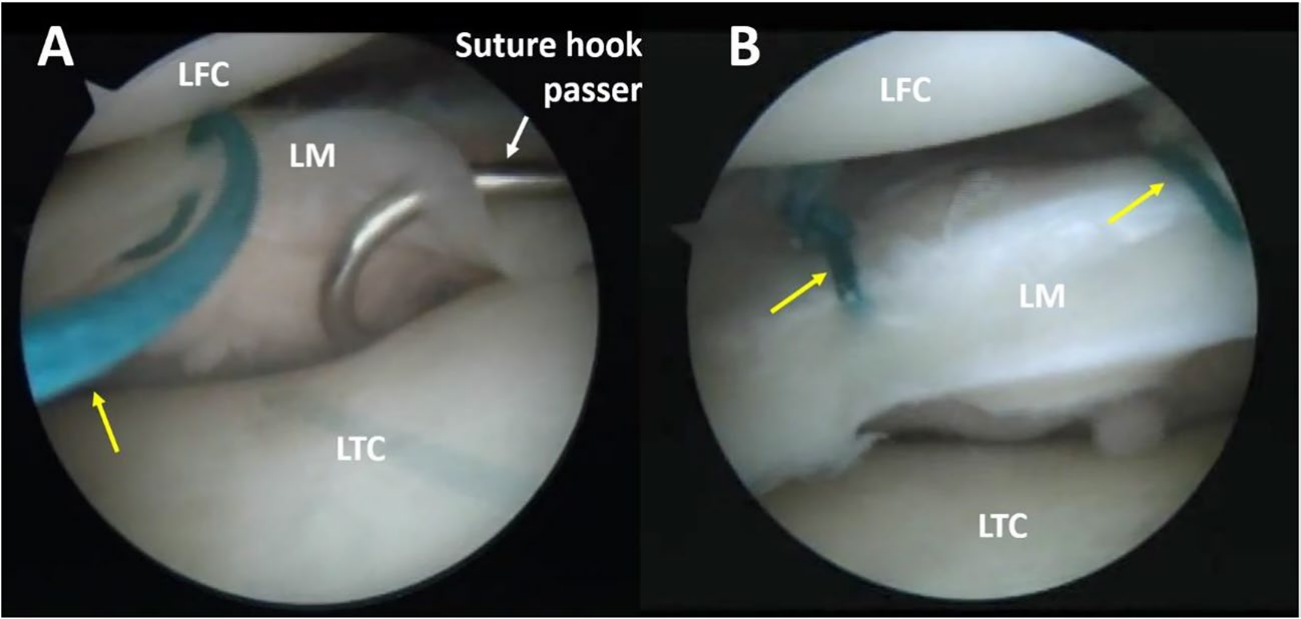
Meniscal repair technique. **A** Passing the suture hook passer through the body of the lateral meniscus can be seen. **B** Tied vertical sutures with knots placed as peripherally as possible. Yellow arrows — non-absorbable Ethibond Excel 2.0 suture. LFC, lateral femoral condyle; LM, lateral meniscus; LTC, lateral tibial condyle

The first stage consisted of an all-inside meniscal repair using suture hook passers and non-absorbable sutures, without medial collateral ligament (MCL) release, as elaborated in the “Methods and materials” section, under the heading Surgical Technique of Meniscal Repair (Fig. 2).

**Fig. 3:**
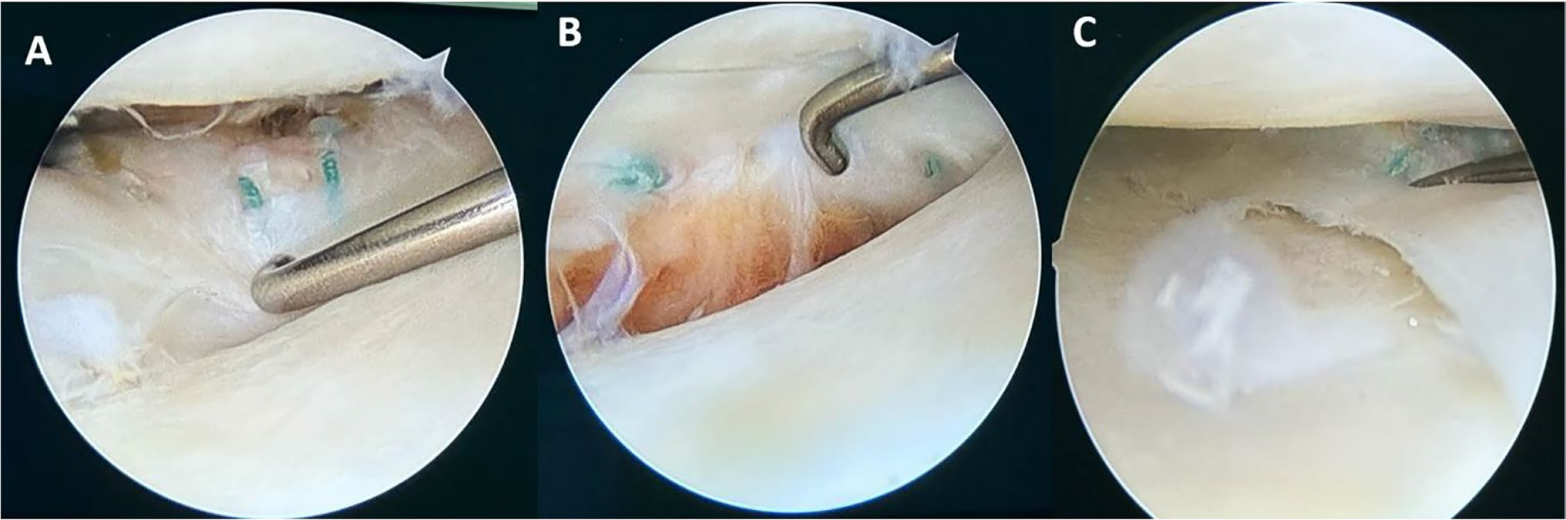
Assessment of meniscal healing during second-look arthroscopy. **A**, **B** Complete healing of both the superior (**A**) and inferior (**B**) surface of the lateral meniscus repair can be seen. **C** Incomplete healing of a medial meniscus repair can be seen with meniscal scar stable, but prone to probing

After six to eight weeks, the second stage surgery was performed, which comprised an ACL reconstruction (ACLR) using quadriceps tendon-bone (QTB) autografts and served as a second-look arthroscopic evaluation of meniscal healing (Fig. 1). The healing of the meniscus around the repair sites was assessed by carefully probing its superior and inferior surfaces. Incomplete healing was defined by (1) meniscal scar that was stable but prone to probing or (2) incomplete fulfilment of the superior or inferior surface of the meniscus with the scar at the sites of repair (Fig. 3). Two operating orthopaedic surgeons performed this assessment independently.

Clinical follow-up was performed at a minimum of 24 months (Fig. 1). Patients who developed recurrent instability prior to the final assessment were excluded from the analysis. Failure was defined by the recurrence of any clinical symptoms and/or symptomatic re-tear of the repaired meniscus. Conversely, success was defined by the absence of clinical symptoms and/or re-tears during clinical assessment.

### Surgical technique of meniscal repair

The surgery was performed under either general or regional anaesthesia. A thigh tourniquet was not used to allow intra-operative observation of bleeding from the meniscus. Routine diagnostic arthroscopy was performed through standard anterolateral and anteromedial portals using a 30° arthroscope. Accessory medial parapatellar and additional posterolateral or posteromedial portals were made to facilitate visualisation and manoeuvrability within the posterior compartments. The diagnosis of medial meniscus (MM) or lateral meniscus (LM) injury was made by direct visualisation and probing with an arthroscopic hook probe.

After confirmation of a complete vertical longitudinal tear of the outer third of the meniscus, it was repaired via a previously described all-inside technique using suture hook passers (Spectrum, Conmed) and three to eight non-absorbable sutures (2.0 Ethibond Excel, Ethicon) depending on the length of the tear [6, 12]. First, the inner and outer margins of the meniscus tear were refreshed using a 30° meniscal rasp (Conmed) with top and bottom serrations. Next, either a left- or right-curved 45° suture hook passer was used to pass a PDS II (Ethicon) suture through the inner and outer parts of the meniscus. The PDS II suture, acting as a shuttle, was then retrieved through corresponding portals outside of the joint by a suture retriever and replaced with the non-absorbable Ethibond Excel 2.0 suture. After placement, the vertical sutures were tied on the outer part of the meniscus using six to seven surgical knots. Careful attention was given when tying the sutures to place each knot as peripherally as possible, to avoid subsequent cartilage irritation and damage (Fig. 2).

### Rehabilitation

For post-operative management, patients were instructed to perform 5 min of passive hyperextension and 5 min of passive flexion every hour for the first two weeks after the meniscus repair surgery. Range of motion (ROM) up to 90° of knee flexion was permitted starting on postoperative day one. During the first six weeks, the patients were limited to walking only while using crutches, with a decrease of walking volume. For the first two weeks, only touch-weight bearing was permitted, which was thereafter progressively increased according to the patients’ tolerance of pain for weeks three to six. No twisting or pivoting movements were allowed. Guided physiotherapy was performed from the second week postoperatively. Symmetric hyper-extension and 120° of flexion had to be achieved before progressing onto the second operative stage.

### Statistical analysis

The data were analysed statistically using Statistica 13.3 software. A two-sided Fisher exact test was used to compare the rate of clinical failure between patients with complete and incomplete healing during second-look arthroscopy. Calculation of relative risk (RR) or odds ratio (OR) was not possible due to 0% clinical failure rate in one of the groups.

## Results

The diagnostic assessment of the first-stage procedure confirmed 15 cases of “bucket-handle” tears, ten of the medial meniscus (MM) and five of the lateral meniscus (LM); 26 cases of posterior horn tears, 16 of the MM and ten of the LM and one case of an anterior horn tear of the LM (in the same patient who also had a posterior horn tear of the MM). In total, there were 26 MM tears and 16 LM tears across the 41 patients. The results of diagnostic arthroscopy are summarised in Table 1.

**Table 1:** Meniscal tear type according to diagnostic part of arthroscopy

	Classification of meniscal tear			
	“Bucket-handle”	Posterior 1/3	Anterior horn	Total
MM	10	16	0	26
LM	5	10	1^a^	16
Total	15	26	1^a^	41^b^

All patients had post-traumatic, longitudinal, vertical meniscal tears

*MM* medial meniscus, *LM* lateral meniscus

^a^The patient with an anterior horn tear of the LM also had a tear of the posterior 1/3 of the MM

^b^The patient with an anterior horn tear of the LM and a tear of the posterior 1/3 of the MM was counted only once in this total for consistency with the total number of patients presented in the Methods section

**Fig. 4:**
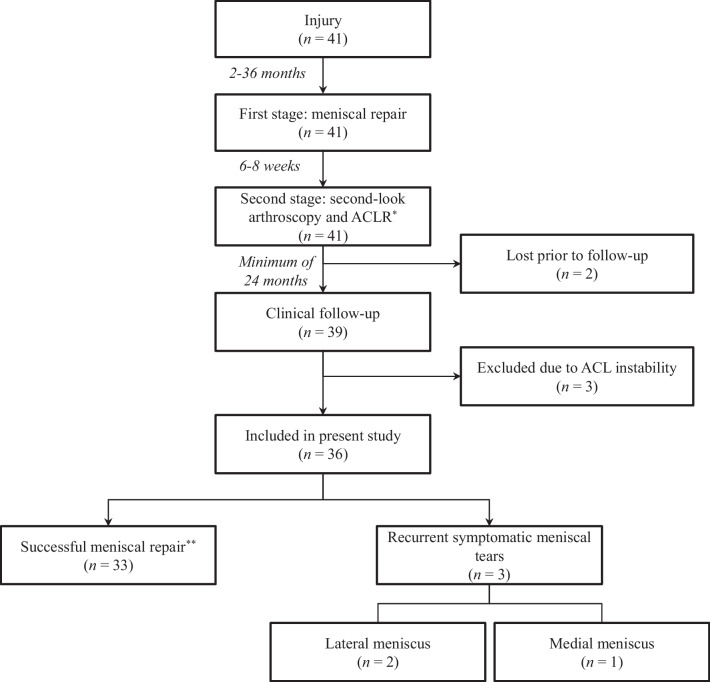
Flowchart of patient outcome scenarios. *31 out of the 41 patients (75.6%) evaluated during second-look arthroscopy showed complete healing of the meniscus repair; ten out of the 41 patients (24.4%) showed partial healing of the meniscus repair. **Saphenous neuropathy occurred in one patient (2.4%)

As summarised in Fig. 4, in the second-look assessment performed during the second stage procedure (ACLR), complete healing of the meniscal repair sites was observed in 31 out of the 41 cases (75.6%). All patients’ menisci were stable in arthroscopic evaluation using surgical probes, even in those whose meniscal repair sites were not completely healed. Two patients were lost prior to follow-up. Among the 39 patients that were available for follow-up (which ranged from a period of 24 to 73 months), three had developed recurrent instability and therefore were excluded from this study. Summarily, there were three out of 36 cases of symptomatic recurrent re-tears (8.3%) during follow-up: one of the MM and two cases of the LM. The time to recurrence ranged from 18 to 28 months. There was one case of persistent saphenous neuropathy (2.4%). The flowchart of patient outcome scenarios is presented in Fig. 4.

All 27 patients within the complete healing group achieved clinical success at the final assessment. In the incomplete healing group, six out of nine patients achieved clinical success at follow-up. The rate of clinical failure at follow-up was significantly higher in patients with incomplete healing during second-look arthroscopy than in patients with complete healing (*p* = 0.012). The relationship between meniscal healing assessed during the second-look arthroscopy and clinical success at follow-up is summarised in Table 2.

**Table 2:** Clinical outcomes in patient groups accordingly to the second-look arthroscopy

		Second-look arthroscopy (6–8 weeks post-op)		
		Incomplete healing	Complete healing	Total
Follow-up (min. 24 months)	Lost/excluded	1	4	5
	Recurrent tears	3	0	3
	Successful repair*	6	27	33
	Total	10	31	41

*Defined as absence of clinical symptoms and re-tear during clinical assessment at follow-up

## Discussion

The most important finding of the present study was that incomplete healing of the meniscus observed during early second-look arthroscopy did not indicate failure of repair at follow-up. There was a significant association between incomplete meniscal healing and the presence of recurrent re-tears of the repaired meniscus. However, six out of the nine patients (66.7%) that exhibited only incomplete meniscal healing during second-look arthroscopy still achieved good clinical results (i.e., absence of clinical symptoms or recurrent meniscal tears). Therefore, it can be concluded that the early arthroscopic finding of incomplete healing does not necessarily result in longer-term failure of repair and does not allow for an easy decision of whether to perform any additional surgical interventions. These results show that while second-look arthroscopy is the gold-standard method for accurate assessment of meniscal healing, eight weeks may be too early to correlate the existence of incomplete healing of the meniscus at the repair site with long-term failure.

The results of this study also demonstrate that in comparison to other methods of meniscal repair, the all-inside suture technique with the use of suture hooks yields successful clinical and functional outcomes. Several other studies have reported comparable clinical success using similar surgical techniques with the use of suture hooks. Ahn et al. [15] assessed repairs of the posterior horn of the medial meniscus using a suture hook and found complete healing in 84.3% of patients. Gousopoulos et al. [16] compared repairs of longitudinal tears of the posterior horn of the MM at the time of ACLR using either an all-inside meniscal repair device or suture hooks. The secondary meniscectomy rate in patients treated with meniscal repair devices was greater than twofold higher. Similarly, Seo et al. [10] described second-look arthroscopic findings of a meniscal posterior horn of MM or LM repair with concomitant ACL reconstruction and demonstrated better healing with a suture hook (82.1%) than with all-inside repair devices (54.5%). Helou et al. [17] reported that out of 61 patients with bucket-handle MM tears who underwent all-inside meniscal repair with suture hooks during primary ACLR, there were nine repair failures (14.8%), which was defined by the number of secondary medial meniscectomies. The clinical outcomes of several studies that had performed meniscal repairs of post-traumatic, longitudinal, vertical, meniscal tears using suture hooks are summarised in (Table 3).

**Table 3:** Clinical outcomes of various studies after performing meniscal repair using suture hook passers

Study	Number of patients (*n*)	Number of successful outcomes (%)	Criteria for failure	Concomitant ACL injury	Type of sutures used	Second-look arthroscopy
This study	36	33 (91.8)	Symptomatic re-tears	+	Non-absorbable	+
Seo et al. [10]	28	25 (89.3)	Residual cleft > 50% of meniscal thickness	+	Absorbable	+
Ahn et al. [16]	39	38 (97.4)	Residual cleft > 50% of meniscal thickness	+	Absorbable	+
Gousopoulos et al. [17]	237	200 (84.4)	Secondary meniscectomy	+	Absorbable	–
Helou et al. [18]	61	52 (85.2)	Secondary meniscectomy	+	Absorbable	–

While previous studies have reported on the success of meniscal repair in the setting of ACLR [18–20], this study reported successful meniscal repair outcomes in a two-stage procedure in which the meniscal repair was performed during the first stage and the ACLR was performed six to eight weeks later during the second stage. Thus, the cohort of patients presented in this study was ACL-deficient post-operatively for six to eight weeks after meniscal repair. Nevertheless, despite this ACL-deficiency, our cohort achieved meniscal complete healing rates of 75.6% evaluated during second-look arthroscopy (6 to 8 weeks post-operatively) and 91.8% at final follow-up clinical assessment (> 24 months post-op.). Our results suggest that meniscal repair, performed as a part of a two-stage surgical procedure, yields comparably successful meniscal healing rates as when meniscal repairs are performed at the same time as ACLR.

We theorised that the comparably successful meniscal healing rate despite two stages and the potential risk associated with instability prior to the second stage surgery could be connected with two possible reasons.

First, during second-stage ACLR, the healing response could be once again promoted due to bone drilling, resulting in the release of growth factors from the bone marrow and haematoma formation [18, 21, 22]. Second, using non-absorbable sutures for repair could allow for a longer time of healing and rebuilding of the repair site due to approximation of the two sites of tear even when the scar is not yet fully durable [23]. Physiological loading without excessive movement of the repair site could increase healing potential due to promotion of mechano-transduction [24].

### Limitations

The limitations of this study were the varying time from injury to surgery and relatively small number of patients included in this study. As to the varying time from injury to surgery, while this limits the homogeneity of the group, it mimics real-life in-patient scenarios. Therefore, in another aspect, it may very well serve to strengthen the validity of the clinical results presented in this study. As to the second limitation, although the present study was limited by its number of subjects, several studies that had performed a similar surgical technique are presented, with comparable successful clinical outcomes. On the other hand, to the extent of the authors’ knowledge, this was also the first study to assess all-inside suture hook meniscal repairs using non-absorbable sutures.

## Conclusions

Incomplete meniscal healing during early second-look arthroscopy after all-inside meniscal repair using suture hook passers and non-absorbable sutures did not necessarily result in longer-term failure in patients with restored knee stability. The described method of meniscal repair was associated with a low rate of symptomatic re-tears and complications.
